# Resistance exercise promotes functional recovery from peripheral nerve injury

**DOI:** 10.3389/fphys.2025.1653032

**Published:** 2025-10-21

**Authors:** Hameed Al-Sarraf, Hind Al Mallah, Abdeslam Mouihate

**Affiliations:** Department of Physiology, College of Medicine, Kuwait University, Kuwait City, Kuwait

**Keywords:** strength training, axonal myelination, catwalk test, toe spread reflex, transmission electron microscopy

## Abstract

Studies on the effect of exercise before peripheral nerve injuries are scarce, with even less attention given to the effects of resistance exercise. In this study, rats were first trained for 10 weeks using a novel resistance exercise system developed in our laboratory, and then they were subjected to either a mild compression or a moderate crush injury of their sciatic nerve. Functional tests, including toe spread reflex, foot positioning, extensor postural thrust, and CatWalk, were carried out at pre- and on selected days post-injury. Animals were sacrificed, and sciatic nerves were collected on the fifth day and the 14th day after the compression and crush-injured rats, respectively. Myelin proteins were analyzed using Western blot, and the morphology and morphometric parameters of the injury site were assessed using transmission electron microscopy (TEM). Nerve compression-injured rats showed no significant difference between resistance-exercised and control rats on either their functional performance, levels of myelin proteins, morphology, or morphometric measurements. On the other hand, in nerve crush injury, the resistance exercise rats performed better in toe spread and extensor postural thrust scores when compared to the injured controls. The TEM revealed that distal segments of crush-injured nerves of the resistance-exercised rats had better morphology compared to those of the crush-injured controls. Our data suggest that resistance exercise prior to a crush injury to sciatic nerve injury led to a better functional recovery, likely through a pro-myelinating effect.

## 1 Introduction

Peripheral nerve injuries affect over one million subjects worldwide every year (Daly et al., 2012). These injuries can lead to sensory and motor deficits and pain, which negatively affect the quality of life of the patients (Wo et al., 2015; Lopes et al., 2022). Therefore, research is focused on exploring ways to lessen the damage and disability and to improve and accelerate recovery after the nerve injury. Regular exercise and physical activity can help prevent or slow the progression of up to 40 chronic diseases, affecting multiple body systems, including the nervous system (Ruegsegger and Booth, 2018). During exercise, the working muscles produce several myokines that suppress pro-inflammatory cytokines and enhance the production of anti-inflammatory cytokines (Petersen and Pedersen, 1985; Calle and Fernandez, 2010; Gleeson et al., 2011; You et al., 2013).

Exercise after peripheral nerve injuries can help in axonal regeneration, production of neurotrophic factors, muscle reinnervation, and improve strength and functional recovery (Maugeri et al., 2021). Furthermore, physical activity has been shown to decrease numbness and pain caused by peripheral neuropathy, leading to better motor abilities and balance (Li and Hondzinski, 2012). Also, balance training and muscle-strengthening exercises were shown to improve the quality of life by decreasing the pain associated with chemotherapy-induced peripheral neuropathy (Dhawan et al., 2020).

Aerobic exercise has been shown to help in functional recovery, axonal regeneration, and muscle atrophy when applied after sciatic nerve injury (Patel et al., 2017; Kong et al., 2022; Ilha et al., 2008). Few studies have explored the effect of resistance exercise on nerve injury. Resistance exercise was shown to enhance functional recovery, axonal regeneration, and muscle atrophy after sciatic nerve injury (Arabzadeh et al., 2022). Exercise can be applied in a rehabilitative manner involving a special exercise regimen for subjects who have already suffered from an injury or in a protective manner, inducing protection through adaptation to exercise performed before suffering an injury. Exploring the protective impact of exercise before peripheral nerve injury is not feasible in humans. Therefore, the protective effect of exercise on nerve injuries can be investigated in animal models. However, studies on the protective effect of resistance exercise before nerve injuries are scarce, even in animal studies (Koop et al., 2023). It has been shown that resistance exercise improves the morphology of the sciatic nerves and prevents muscle atrophy when conducted before sciatic nerve injury (Kakihata et al., 2016; Malanotte et al., 2017). In this study, we aimed to identify whether adaptation to resistance exercise before nerve injury is supportive of the body’s response to recover from nerve injury. Using a newly developed system for resistance exercise in rats (Al-Sarraf and Mouihate, 2022), we explored whether resistance exercise performed before sciatic nerve injury in rats was protective using two types of injuries to the sciatic nerve with different intensities assessed separately, namely, a mild compression injury and a moderate crush injury. We conducted several motor behavioral tests including toe spread reflex (TSR), foot positioning, extensor postural thrust (EPT) and gait, assessed myelin protein levels, and morphology and morphometrics of the axonal myelination in the control and resistance-exercised rats.

## 2 Materials and methods

### 2.1 Animals

Sixty male Sprague Dawley (SD) sedentary rats, housed in plexiglass cages since birth (∼8 weeks old), were randomly divided into two groups (30 rats/group): the resistance exercise and the control animals. They were weighed weekly. To minimize the number of animals used in this study, each rat was also subjected to a sham injury on the right leg and a sciatic nerve injury on the left leg (Oñate et al., 2016). Sciatic nerve injury was either a mild sciatic nerve compression (SNC) or a moderate sciatic crush injury (SCI). The rats were kept at an ambient temperature of ∼23 °C under a 12-h light-dark cycle (lights on from 8:00 p.m. to 8:00 a.m.) with free access to pelleted chow and water. Ethical approval was obtained from the Health Sciences Center Ethics Committee of Kuwait University, and the rats were treated in accordance with guidelines on the humane handling of experimental animals.

### 2.2 Resistance exercise

A newly developed resistance exercise system was used (Al-Sarraf and Mouihate, 2022). Briefly, using a tunnel and a pulley system, the rats were induced to pull a pre-determined weight tied to their tail. Rats were trained three times per week for 10 weeks by increasing the pulling load progressively and the number of runs at each training session, as well as weekly. The resistance exercise protocols were performed during the animals’ dark cycle in a dark room with a small, dim light. The control animals walked in the tunnel in a similar manner to the resistance exercise group without applying any load.

### 2.3 Sciatic nerve compression

**FIGURE 1:**
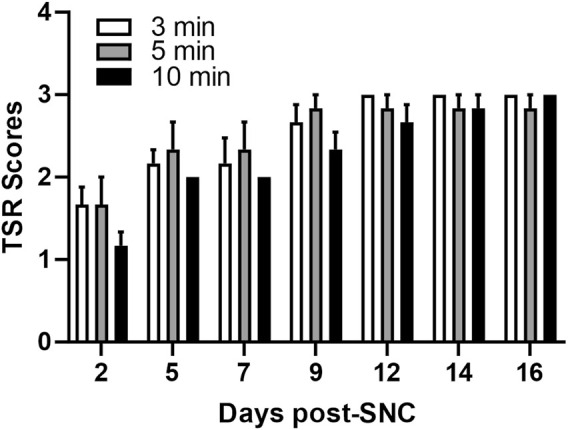
Impact of the duration of sciatic nerve compression on the toe separation reflex. Toe spread reflex (TSR) scores following sciatic nerve compression injury of different durations (3, 5, and 10 min). The normal TSR score for the uninjured limb is 3. Data are presented as mean ± SEM (n = 6 rats/group).

A standard aneurysm clip (Yasergil Phynox, FE-753K) was used to induce SNC. According to the manufacturer, this clip produces a constant 70 g/mm^2^ force. To find a proper compression duration, the sciatic nerve compressions at ∼1 cm away from the greater trochanter were performed for either 3, 5, or 10 min (6 rats/group). The Functional recovery was assessed using the toe spread reflex (TSR) test on days 2, 5, 7, 9, and 12 post-SNC. TSR scores showed no significant difference between 3- and 5-min compressions, with animals appearing fully recovered by day 9 (Figure 1). In contrast, 10-min compression produced a more distinct recovery pattern, but full recovery was delayed until day 12. Accordingly, 10 min of compression and day 5 post-SNC were selected for further analysis.

### 2.4 Sciatic nerve crush

A 12 cm stainless-steel micro-mosquito haemostatic forceps with a serrated straight tip (1.3 mm in width) at three clicks was used to crush the sciatic nerves for 60 s, as this crush duration was used previously (Altun and Kurutaş, 2016; Renno et al., 2015). The site of injury was at ∼1 cm from the greater trochanter to the midshaft and ∼0.5 cm caudally to avoid the trifurcation of the sciatic nerve (Savastano et al., 2014). We used the TSR test daily during the first week to confirm that sciatic nerve crush produced consistent motor deficits suitable for monitoring recovery. All animals showed clear TSR impairment.

### 2.5 Animal surgery

Twenty-four hours after the last resistance exercise or walking with no load through the tunnel, the animals were anesthetized intraperitoneally with ketamine 50 mg/kg (Rotexmedica, Germany) and xylazine 5 mg/kg (Interchemie Company, Holland), and subcutaneously injected with antibiotic Enrofloxacin 5% (T Special LTD., United Kingdom). Ointment Tobrex (Novartis, Spain) was applied to the eyes to prevent dehydration, and both thighs were shaved using an electric shaver. The rat was placed on its side with a rectal probe on a heated pad to maintain the body temperature during the surgery. All surgical tools were sterilized in an autoclave before surgery. The skin was cleaned under the femur bone with betadine and 70% ethanol. Using a surgical blade, the muscles below the femur bone were dissected, and the sciatic nerve was exposed and cleaned of the surrounding tissues. On the left leg, the compression groups received SNC using the aneurysm clip, while the crush groups received SCI using micro-mosquito haemostatic forceps. A few drops of sterilized saline were applied, and the muscles were sutured. The skin was wiped with sterilized H_2_O_2_ (6%) and closed with 9 mm autoclips or sutured. On the right leg, sham surgery was done following the same procedure but without applying any compression or crush injury to the nerve.

### 2.6 Assessment of motor functions

To assess motor functions, toe spread reflex (TSR), foot positioning, extensor postural thrust (EPT), and CatWalk tests were performed on all rats (Renno et al., 2017; Dayawansa et al., 2014; Parvathy and Masocha, 2013). Briefly, the TSR test was performed by holding the rat from its tail and lowering it to a surface to check for toe separation while hanging and on the surface (Renno et al., 2017). This test was evaluated using a three-point scale where score 1 corresponds to no separation of toes, which is the least functional, score 3 corresponds to full separation of the toes, indicating a fully functional toe reflex, and score 2 corresponds to anything in between.

For both SNC and SCI, the motor function tests were performed 1 week pre-surgery. For the SNC, the motor functions were tested on the second and fifth days post-injury. In contrast, the SCI resulted in larger damage for which the recovery took a longer time. In a pilot study we observed a partial recovery by the eighth and the 12 days post-SCI. For this reason, we tested motor functions for a longer period of time lapsing from the 2nd until the 12th days post-SCI to account for the longer recovery period in the SCI model.

This test was performed at 1 week pre-surgery, second, and fifth days post-SNC model, and for the SCI, it was performed at 1 week pre-surgery, 2nd, 5th, 8th, and 12th days post-injury.

A foot positioning test was also used to assess the position and posture of the feet of the rat while standing and walking (Renno et al., 2017). This test was also evaluated using a three-point scale, where score 1 means full eversion, score 2 is mild eversion, and score 3 is when the foot is fully supported on the surface, indicating fully functional foot positioning. This test was done on the same selected time points that were used for the TSR test.

To assess the power of the hind limbs, the EPT test was performed. This test was done by holding the rat with a piece of cloth upright and placing their foot on an open digital scale (Dayawansa et al., 2014). The measurements were obtained in grams corresponding to the force of the lower limb. This test was also done on the same selected time points that were used for the TSR and foot positioning tests. For these three functional tests, an average of three measurements was taken for each leg. Rats’ gait was monitored using the CatWalk gait analysis system (Noldus Information Technology, Netherlands) (Parvathy and Masocha, 2013). The captured videos were analyzed using CatWalk software version 7.1. Parameters such as the maximum contact area, intensity of contact, print area, standing phase, and swinging phase of the hind paws were measured (Parvathy and Masocha, 2013). This test was done 1 week pre-surgery, 2 and 5 days after SNC, and at 1 week pre-surgery, 5 and 12 days after SCI.

### 2.7 Tissue collection

Rats were anesthetized with urethane 1.5 mg/kg (Sigma Life Science, China) intraperitoneally and perfused with ice-cold PBS. The sciatic nerves were collected on the fifth day post-SNC (Figure 1) and the 14th day post-SCI, as shown in previous studies (Renno et al., 2017; Ramli et al., 2017; Wang et al., 2018). The intact and injured sciatic nerves with a length of ∼1 cm at the site of injury were extracted, snap-frozen in liquid nitrogen then stored at −80 °C for the Western blot experiment. The site and distal regions (∼1–2 mm) of the injured sciatic nerve were collected and fixed by immersion in 3% glutaraldehyde and Millonig’s phosphate buffer for the transmission electron microscopy (TEM) study.

### 2.8 Western blot

Nerve samples were added to a solution of lysis base buffer with a protease inhibitor tablet (Roche Applied Sciences, Germany) and homogenized using Next Advance Bullet Blender Storm 24 (USA), then centrifuged at 12,000 rpm for 15 min at 4 °C. The supernatant was collected, and the protein levels were assayed using the bicinchoninic acid (BCA) assay. The proteins were separated using the SDS-PAGE technique using a 15% acrylamide gel. Semi-dry transfer was used to transfer the proteins from the gel to a nitrocellulose membrane (Bio-Rad company, Germany). After the transfer and blocking in 5% fat-free dry milk, the membrane was washed and incubated with primary antibodies overnight at 4 °C. The membranes were incubated in a primary monoclonal mouse anti-myelin basic protein antibody (anti-MBP, Calbiochem, 1:5000) or monoclonal mouse anti-phospho-MBP antibody clone P12 (anti-pMBP, Merk KGaA Darmstadt, Germany, 1:3000). After washing, the membranes were incubated with horseradish peroxidase-conjugated (HRP) donkey anti-mouse IgG (H + L) secondary antibody (Invitrogen, 1:2000) for 2 h at room temperature. Beta actin polyclonal rabbit primary antibody was used as a house-keeping protein (Invitrogen, 1:5000), followed by donkey anti-rabbit IgG secondary antibody (Invitrogen, 1:5000). The membranes were then exposed to an enhanced chemiluminescent solution (clarity western ECL substrate; Bio-Rad, USA), and the immunoreactive bands (IR) were digitally detected using Azure Biosystems scanner (USA). Optical density of the protein bands was measured using Azure Analysis Software version 2.0.028. The ratios of the OD of each protein to that of actin were calculated and used as a semi-quantitative measure of protein levels.

### 2.9 Transmission electron microscopy

Sciatic nerves were dehydrated using increased concentrations of ethanol and embedded in epoxy resin. Ultra-thin cross sections (100 nm) were cut from the resin blocks and mounted on copper grids. The sections were then stained with uranyl acetate and lead citrate. Images were acquired using a 1500X objective using JEOL’s JEM-1200 EXII, Transmission Electron Microscope (Tokyo, Japan). The morphometric parameters studied were focused on the myelinated axons, including the number of myelinated axons, myelin thickness, and g-ratio. TEM Images were taken from the sciatic nerves of SNC (3 per rat, 3 rats per group) and SCI rats (5 per rat, three rats per group). For each rat, the images were taken from the sham sciatic nerve, and at two sites of the injured sciatic nerve: at the site of injury, and ∼1–2 mm distal to the site of injury. Therefore, a total of 9 (SNC model) to 15 (SCI model) images were taken. The number of myelinated axons was counted per image using Fiji ImageJ software. The mean diameter of axons of the myelinated fibers (d) in µm and the mean diameter of the whole myelinated nerve fibers (D) in µm were calculated automatically using Image Pro Plus version 10 analysis software (Media Cybernetics, Rockville, MD, USA), which is commonly used to analyze TEM nerve cross-sectional images (Renno et al., 2017; Ronchi et al., 2023). From the mean diameters, we calculated the myelin thickness as (D-d/2) and the g-ratio as (d/D). A smaller value of the g-ratio indicates better myelination of the nerve fiber. The number of myelinated axons and the average of each parameter per image were averaged by rat. Comparison was done between each injured group (site of injury or distally) with its corresponding sham group, and between exercised and control rats at each studied area. For the SNC model, a total of 2079 axons were analyzed for myelin parameters with a range of around 210–482 axons/group. For the SCI model, a total of 3278 axons were analyzed for myelin parameters with a range of around 280–703 axons/group.

### 2.10 Statistical analysis

Statistical analysis was performed using SPSS (version 25.0). All behavioral data and body weight were analyzed using repeated measures ANOVA followed by an adjusted Bonferroni *post hoc* test. The data from Western blot and TEM were analyzed using a two-way ANOVA followed by a Bonferroni multiple comparison test. Statistical significance was considered when (p-value <0.05). Data were presented as mean ± SEM.

## 3 Results

### 3.1 Functional motor tests after sciatic nerve injury

**FIGURE 2:**
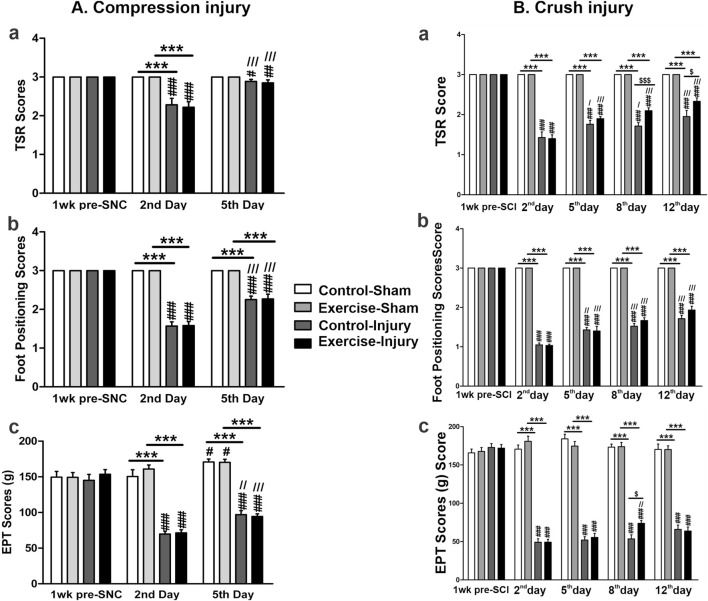
Functional motor tests in resistance-exercised and control rats after sciatic nerve injury. The tests related to sciatic nerve compression (SNC) are shown in panel **(A)**. Measurements were taken at baseline (pre-injury) and on days 2 and 5 post-SNC to evaluate the toe-spread reflex [TSR, **(Aa)**], foot positioning **(Ab)**, and extensor postural thrust [EPT, **(Ac)**]. Tests related to sciatic crush injury (SCI) are shown in panel **(B)**. Measurements were taken at baseline and on days 2, 5, 8, and 12 post-SCI to assess the same parameters [TSR, **(Ba)**]; foot positioning. **(Bb)**; EPT, **(Bc)**. For both panels, the sham indicates the uninjured contralateral sciatic nerve of each animal. Data are expressed as mean ± SEM (n = 19–20 rats per group). Statistical symbols: * compared with sham, # compared with pre-injury,/compared with day 2, and $ indicating exercised *versus* control injured rats. (#,/, $ p < 0.05; ##,//p < 0.01; ***, ###,///, $$$ p < 0.001).

To assess the impact of resistance exercise on peripheral nerve recovery, we applied two types of nerve injuries to the sciatic nerve: the sciatic crush injury (SCI) and the sciatic nerve compression (SNC). As shown in Figure 2A, the SNC resulted in significantly lower scores for TSR (Figure 2Aa), foot positioning (Figure 2Ab), and EPT (Figure 2Ac) in both the control and exercised rats on the second and fifth day post-SNC when compared to the scores in their respective sham-treated legs. A slight, but significant improvement was observed in these three scores on the fifth day post-SNC injury that was not dependent on the resistance exercise but was probably due to the naturally occurring recovery. Similar effects were noticed when the sciatic nerve was subjected to the SCI, as seen in Figure 2B. While there was normal improvement of these scores over time, ranging from day 2 to day 12 post-SCI injury, we observed that resistance exercise enhanced these recoveries, particularly in TSR (Figure 2Ba) and EPT (Figure 2Bc) scores on day 8, and in TSR scores on day 12.

**FIGURE 3:**
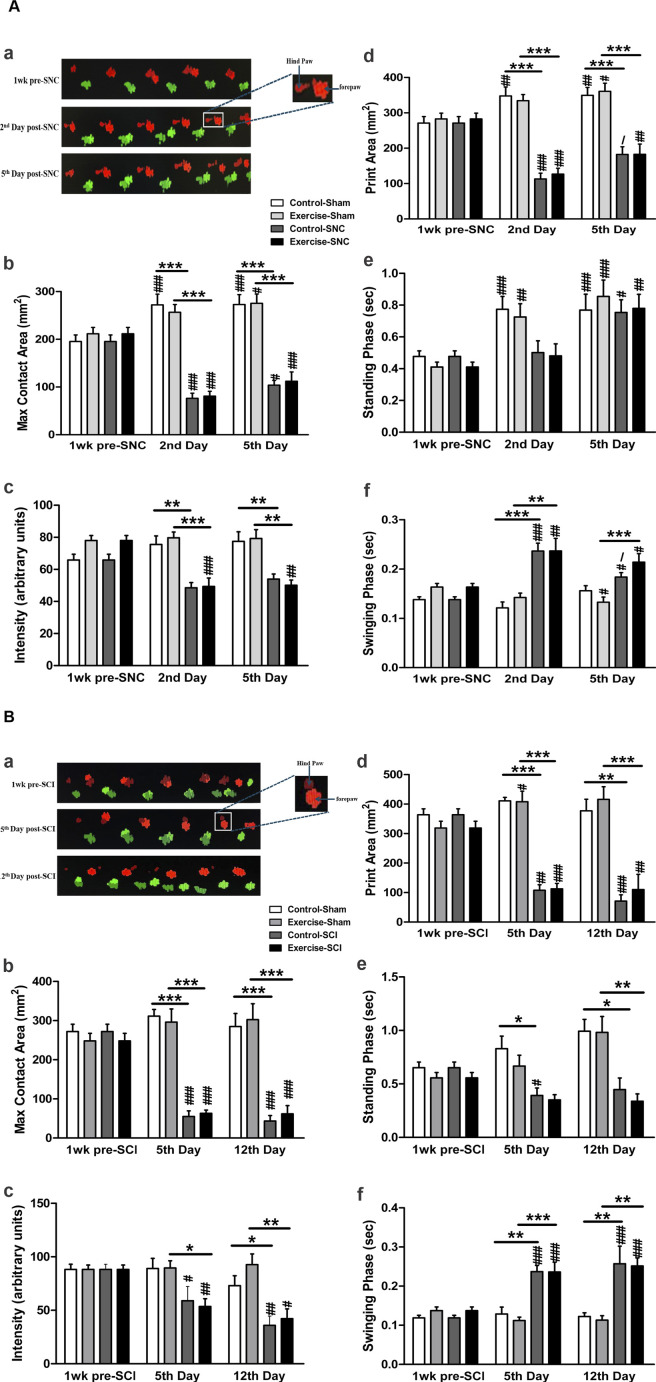
CatWalk gait analysis in resistance-exercised and control rats following sciatic nerve injury. The CatWalk gait analyses related to sciatic nerve compression (SNC) are shown in panel **(A)**. The micrograph at the top displays the walking steps **(Aa)** across the CatWalk apparatus (right to left). The injured limb on the left (red) and the contralateral sham-operated limb (green) were assessed 1 week before SNC and on days 2 and 5 post-SNC. The graph bars depict the maximum contact area **(Ab)**, the intensity of paw contact **(Ac)**, the print area during full stance **(Ad)**, the stance phase duration **(Ae)**, and the swing phase duration of the hind paws **(Af)**. The CatWalk gait analysis related to sciatic crush injury (SCI) is shown in panel **(B)**. The graph bars illustrate the maximum contact area **(Bb)**, paw contact intensity **(Bc)**, print area during full stance **(Bd)**, stance phase duration **(Be)**, and swing phase duration of the hind paws **(Bf)**. Data are presented as mean ± SEM (n = 7–10 rats/group). In both panels: * indicates comparison with sham, # indicates comparison with pre-injury, and/indicates comparison with day 2 in SNC or day 5 in SCI. (*, #,/p < 0.05; **, ##p < 0.01; ***, ###p < 0.001).

The gait steps were analysed using the CatWalk system (Figure 3). Following SNC, both control and exercised rats showed significantly lower maximum contact areas (Figures 3Aa, Ab), step intensity (Figure 3Ac), and print areas (Figure 3Ad) of the hind paws compared to their corresponding sham-operated leg on the second and the fifth day. However, the standing phase was not significantly affected by the compression in either control or exercised rats on either the second or the fifth day after SNC (Figure 3Ae). The swinging phase was longer in both the control and exercised rats when compared to their corresponding shams on the second day post-SNC (Figure 3Af), while on the fifth day after the compression, only the exercised rats showed significantly longer swinging durations compared to their sham. Resistance exercise did not significantly improve any SNC-induced alteration of these gait parameters.

As shown in Figure 3B., SCI significantly lowered the maximum contact area (Figures 3Ba, Bb), the intensity of the contact of the hind paws (Figure 3Bc), and print area (Figure 3Bd), and increased the swinging phase (Figure 3Bf) in both exercised and control rats when compared to their corresponding shams on the 5th and 12th days. Compared to their corresponding shams, the standing phase was significantly reduced on the fifth day after SCI in the control rats, and on the 12th day in both control and exercised rats (Figure 3Be). As was seen with SNC injury, resistance exercise did not significantly improve any SCI-induced alteration of these gait parameters (p > 0.05).

### 3.2 Resistance exercise and the basic myelin proteins in sciatic nerve injury

**FIGURE 4:**
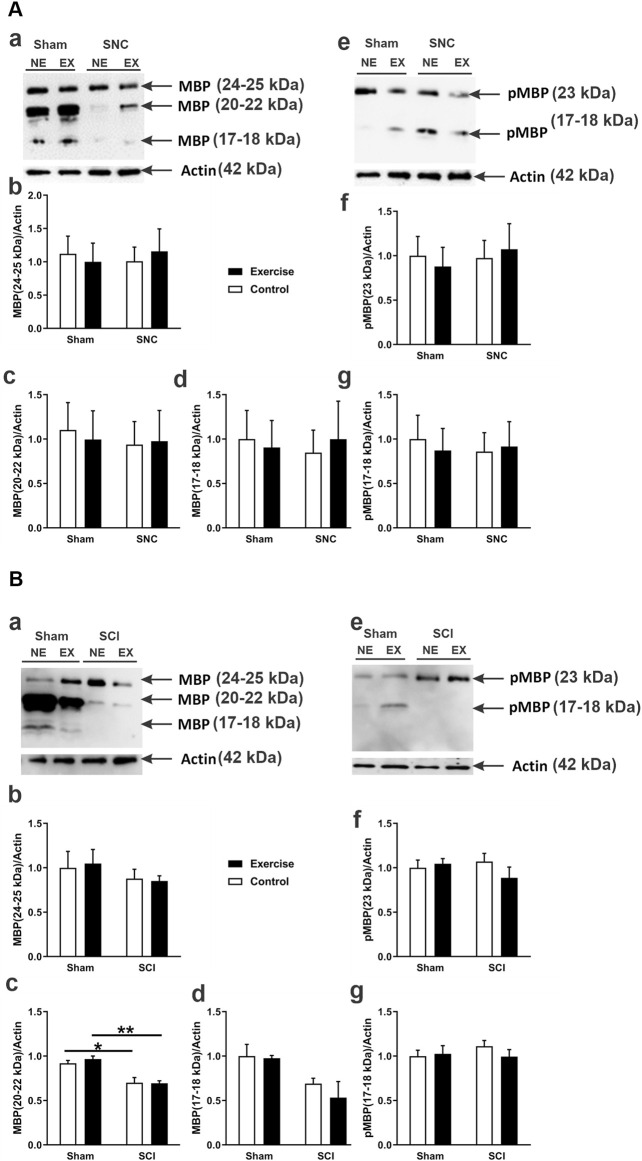
MBP and phosphorylated MBP (pMBP) protein expression following sciatic nerve injury in exercised and non-exercised rats. Panel **(A)** displays Western blot data related to sciatic nerve compression (SNC). The micrograph in **(Aa)** shows a representative Western blot illustrating different MBP isoforms expressed in the sciatic nerve of exercised (EX) and non-exercised (NE) rats that were either sham-operated or subjected to SNC. The graphs in **(Ab–d)** present semi-quantitative analyses of these MBP isoforms. The micrograph in **(Ae)** shows a representative Western blot of pMBP isoforms. The graphs in **(Af,g)** provide semi-quantitative analyses of pMBP isoforms (n = 7–9 rats/group). Panel **(B)** displays Western blot data about sciatic crush injury (SCI). The micrograph in **(Ba)** shows a representative Western blot of various MBP isoforms expressed in the sciatic nerve of exercised (EX) and non-exercised (NE) rats that were either sham-operated or subjected to SCI. The graphs in **(Bb–d)** show semi-quantitative analyses of those MBP isoforms. The micrograph in **(Be)** presents a representative Western blot of pMBP isoforms. The graphs in **(Bf,g)** show semi-quantitative analyses of pMBP isoforms (n = 4 rats/group). Data are expressed as mean ± SEM. *p < 0.05, **p < 0.01.

Levels of MBP and pMBP at the site of the SNC on the fifth day post-compression injury are shown in Figure 4A. The compression had no significant effect on the levels of either MBP or pMBP isoforms at the injured nerves in both the control and exercised rats when compared to their corresponding shams. Also, there was no significant difference in these myelin levels between the injured exercised and control rats.

In contrast, the SCI (see Figure 4B) resulted in a significant reduction in the levels of an isoform of MBP with an apparent MW of 20–22 kDa in both the control and exercised rats when compared to their corresponding sham-operated legs (Figure 4Bc). However, the injury had no significant effect on the levels of any pMBP isoform in the crush-injured nerves of either control or exercised rats when compared to their corresponding shams (Figures 4Be, Bf, Bg). Resistance exercise did not significantly affect the expression levels of either MBP or pMBP in the crush-injured sciatic nerve.

### 3.3 Resistance exercise and the myelin structure in the injured sciatic nerve

**FIGURE 5:**
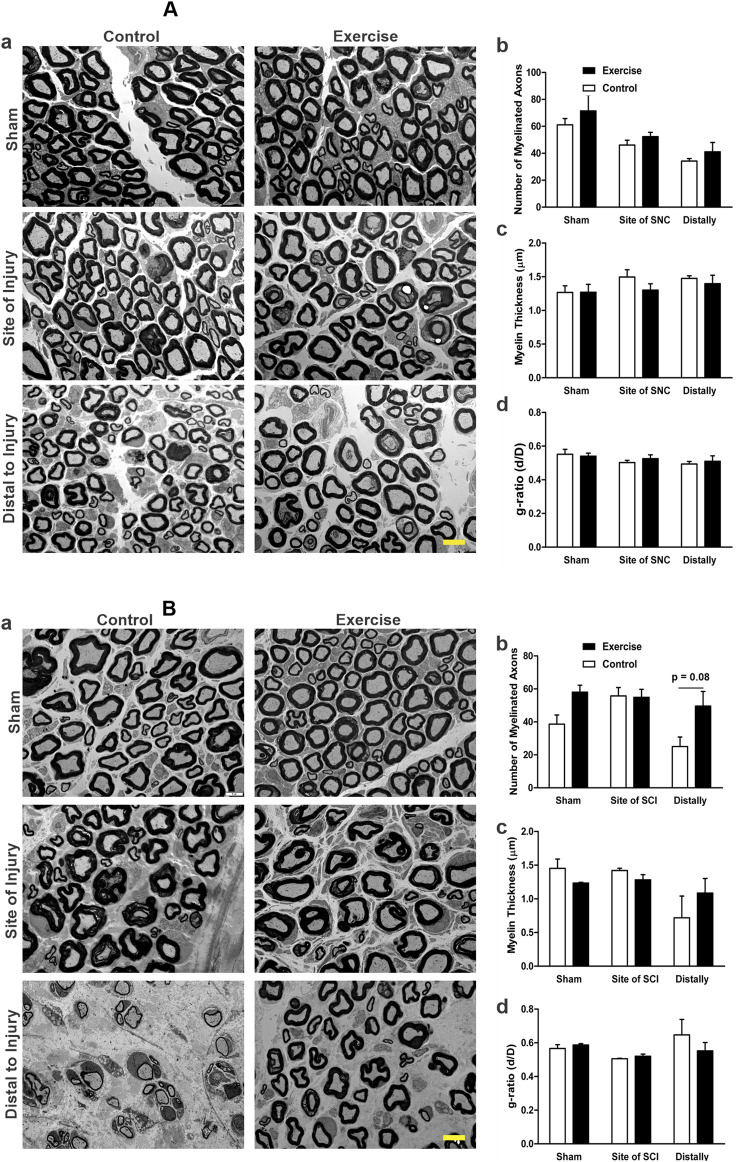
Effect of resistance exercise on sciatic nerve morphology and morphometrics following injury, assessed by TEM. Panel **(A)** shows the transmission electron microscopy (TEM) data related to sciatic nerve compression (SNC). Representative TEM images of sciatic nerve cross-sections from sham, injury site, and 1–2 mm distal regions in control and resistance-exercised rats are shown in **(Aa)**. The graphs show the number of myelinated axons **(Ab)**, myelin thickness **(Ac)**, and the g-ratio **(Ad)**. The numbers of analyzed axons were: 448 (Control-Sham), 482 (Exercise-Sham), 332 (Control-Site), 374 (Exercise-Site), 210 (Control-Distal), and 233 (Exercise-Distal). Panel **(B)** shows the transmission electron microscopy (TEM) data related to the sciatic crush injury (SCI). Representative TEM images of sciatic nerve cross-sections from sham, injury site, and 1–2 mm distal regions in control and resistance-exercised rats are shown in **(Ba)**. The graphs show the number of myelinated axons **(Bb)**, myelin thickness **(Bc)**, and the g-ratio **(Bd)**. The numbers of analyzed axons were: 459 (Control-Sham), 703 (Exercise-Sham), 624 (Control-Site), 639 (Exercise-Site), 280 (Control-Distal), and 573 (Exercise-Distal). Data are expressed as mean ± SEM (n = 3 rats/group). Scale bar = 5 μm.

The transmission electron microscope images of sciatic nerves 5 days after SNC show that both the control and exercised shams display normal thick myelin sheath around their axons with occasional scattered unmyelinated axons (Figure 5Aa). Resistance exercise did not appear to affect the morphology, density, and distribution of the myelinated axons of uninjured sham sciatic nerves. Compared with their corresponding shams, the nerve fibers subjected to SNC in both control and exercised rats showed slightly thinner and irregular myelin sheaths with intra-axonal debris, which is more apparent in the areas distal to the site of compression (Figure 5Aa). Resistance exercise did not significantly alter the number of myelinated axons (Figure 5Ab), myelin thickness (Figure 5Ac), and myelin g-ratio (Figure 5Ad) in the sciatic nerves subjected to the compression injury.

Fourteen days after SCI, both exercised and control rats showed damage features at the site of injury and distally when compared to the regular myelinated axons with thick, compact myelin sheaths seen in the shams (micrographs in Figure 5Ba). Injured nerves exhibited irregular, loosely compacted myelin sheaths with intra-axonal debris. Compared to the control, resistance-exercised crush-injured sciatic nerves showed a similar pattern in the morphology of damage at the site of injury, but showed a better morphology with greater density of myelinated fibers and thicker myelin sheaths at the distal areas (micrographs in Figure 5Ba). Morphometric analysis of the micrographs showed no significant difference in the number of myelinated axons (Figure 5Bb) and myelin thickness (Figure 5Bc) of injured nerves at the site of injury for both the control and exercised rats when compared to their corresponding sham-operated legs. Although statistical comparison of the number of myelinated fibers distally did not reach significance (p = 0.08), visual inspection of electron micrographs revealed a greater myelinated fiber density in the resistance-exercised group compared to the non-exercised one (Figure 5Bb and micrographs in Figure 5Ba).

## 4 Discussion

In the current study, we investigated whether resistance exercise before sciatic nerve injury can protect against injury and improve recovery from injury. We provided evidence that 10 weeks of resistance exercise before sciatic nerve injury exerted some protective effects manifested by a partial functional recovery and better nerve morphology than the control. This protective effect of resistance exercise could be detected when the sciatic nerve was crushed but not when it underwent a milder compression.

We used a tunnel and pulley resistance exercise system in rats, which was developed in our laboratory (Patent No.: US 10,426132 B2) (Al-Sarraf and Mouihate, 2022). The main goal of developing such a system was to induce rats to perform resistance exercise with minimal stress, suitable for studying the effect of resistance exercise on the nervous system. An important aspect of our system is that to induce resistance exercise, no negative or positive reinforcements were required, unlike other experimental models of resistance exercise (Baar and Esser, 1999; Lee et al., 1985; Hornberger and Farrar, 2004; Silveira et al., 2011; Nicastro et al., 2012; Łochyński et al., 2016). With our resistance exercise system, there is no direct handling of the animal during the exercise. In comparison, other models of resistance exercise require repeated handling and occasional electric shock, which can be counterproductive when studying the beneficial effects of exercise on the nervous system. Previously, using this tunnel and pulley resistance exercise system on healthy adult rats for 10 weeks, we observed skeletal muscle hypertrophy (Al-Sarraf and Mouihate, 2022) and increased hippocampal neurogenesis (data not shown).

In the current study, we used the resistance exercise system to train rats for 10 weeks and then applied two injury models using different intensities of compression forces on the sciatic nerve. A group of rats had a mild sciatic nerve compression injury using a clip of 70 g/mm^2^ force, and another group received a more severe injury by crushing the sciatic nerve using micro-mosquito forceps. Days after injury, using several motor behavioral tests, the functional performances of rats were evaluated. Also, the injured sciatic nerves were assessed for the levels of myelin proteins and morphological changes.

The mild compression injury to the sciatic nerve was induced by a clip of 70 g/mm^2^ force for 10 min. This duration of compression showed the strongest lasting effect of injury assessed by the behavioral motor test, TSR (Figure 1). Previous studies had shown that a compression force of 60 g/mm^2^ for 10 min on the sciatic nerve led to complete paralysis on the first day post-injury, which was associated with a drastic functional motor recovery by day 7 post-injury, decreased myelin thickness, and increased endoneural space from day 1–7 (Omura et al., 2004). This is comparable to our sciatic nerve compression model, as we also observed functional motor deficit by the second day, with some recovery by the fifth day. Also, on the fifth day post-compression injury, EM revealed thin and irregular myelin sheaths with intra-axonal debris. However, we could not detect any significant change in the levels of MBP/pMBP after the mild compression. This could be either because the injury was not strong enough to induce detectable changes in the assessed myelin proteins or because the selected fifth day for sacrifice allowed enough time for myelin recovery from the compression injury. Also, it seems that the mild compression resulted in a prompt recovery, so we were not able to detect noticeable differences between the control and exercised rats. On the other hand, the crush model caused a more severe injury, as manifested with deterioration in motor function similar to findings observed in previous studies (Renno et al., 2017; Ramli et al., 2017). The sciatic nerve crush revealed that crush-injured resistance-exercised rats exhibited significant improvement in the functional motor tests, TSR, and EPT compared to the crush-injured controls. Kakihata *et al.* (2016) subjected rats to aquatic jumps with loads for 20 days before and after the sciatic nerve crush for 30 s and did not observe any protective effects on the functional performance (Kakihata et al., 2016). This apparent discrepancy is likely due to the difference in the duration and the mode of resistance exercise (Kakihata et al., 2016). To the best of our knowledge, this is the first study exploring the protective effect of resistance exercise before sciatic nerve injury. Other studies have used ladder climbing with loads in a rehabilitative manner after rat sciatic nerve crush injury and showed improved performance in some of the functional tests (Arabzadeh et al., 2022).

We further evaluated the extent of crush injury/recovery by assessing the levels of MBP and pMBP proteins in the injured sciatic nerves since MBP and its phosphorylated (pMBP) form are the major hallmarks of myelination recovery of the nervous system. Compared to the compression injury, the crush injury showed significantly lower levels of MBP when compared to those seen in the uninjured sham. These data are in line with the results reported previously after sciatic nerve crush (Renno et al., 2017) and ligation injury (Setton-Avruj et al., 2002). However, our resistance exercise training for 10 weeks before the sciatic nerve crush did not have a significant effect on the levels of either MBP or its phosphorylated form. To our knowledge, there are no previous studies on the effect of resistance exercise on myelin protein levels. This compels further investigations exploring longer duration of exercise, earlier or later than the 14th day sacrifice point post-injury, and assessment of other proteins such as myelin protein zero (P0). Previously, it was shown that 4 weeks of rehabilitative swimming in rats after ligation injury enhanced the expression of myelin P0 (Ghanbari et al., 2023).

Nerve morphology by electron microscopy revealed slight nerve damage at the site of injury and distally 5 days after the compression injury. However, there was no significant difference between the exercised rats and the controls. Also, no significant differences were detected between the exercised and control groups in the morphometric measurements, including the number of myelinated axons, myelin thickness, and g-ratio after the compression injury. Using a similar mild compression of the sciatic nerve, Omura et al. observed features of thin myelinated axons at the site of compression, with most of the axons and myelin sheaths having normal features 30 mm distal to the compression site (Omura et al., 2004). Like our observation, they also showed that mild compression of the sciatic nerve exerted no significant effect on the number of (axonal) nerve fibers (Omura et al., 2004). However, 10 weeks of resistance exercise before the crush injury of the sciatic nerve resulted in better morphology of the sciatic nerve at the distal segments of the exercised rats compared to the distal segments of the crush-injured control rats. On the other hand, there was no significant effect on the morphometric measurements of myelin thickness and g-ratio between the two groups. Similar findings in morphology and morphometry after sciatic nerve crush injury in resistance exercise-trained rats for 20 days were reported by Kakihata et al. (2016). Despite not reaching statistical significance due to small sample size (3 exercise, 3 control), the observed doubling in the number of myelinated fiber density and consistent pattern across all inspected EM images strongly supports a biologically relevant effect of resistance training. The clear visual distinction between groups supports this interpretation. While the number of animals is relatively small, which could have limited the statistical power, this type of EM study is laborious, as a large number of axons needed to be analyzed. In the present study, we analyzed more than 3000 axons with an average of 540 axons per rat group.

## 5 Conclusion

Clinicians often undertake management of the nerve injury through medication and/or rehabilitative exercises. The majority of investigations on the effect of exercise on peripheral nerve injuries focus on rehabilitation from the injury, since investigating the protective impact of exercise before nerve injury is not feasible in humans. Also, most animal research in this field is focused on the effect of aerobic exercise, with minimal attention given to the possible benefits of adaptation to resistance exercise before injury. In this study, we used a suitable resistance exercise system to explore the possible protective effect of exercise on sciatic nerve injury. We showed that 10 weeks of resistance exercise was enough to provide some protection for the crush-injured sciatic nerve. However, taking this study further requires taking into consideration several important parameters, as they have a major influence on the outcome of adaptation to exercise. The type of exercise, intensity, frequency, volume, and duration are variables that need further investigation. Furthermore, the injury and its severity, and the time given for recovery, can reveal important evidence in this field. In this study, animals were trained 3 times per week for 10 weeks with progressively increasing loads and runs. Changing these parameters may also change the effectiveness of the exercise.

## Data Availability

The original contributions presented in the study are included in the article/supplementary material, further inquiries can be directed to the corresponding author.
