# Supplementary material for: Resistance exercise promotes functional recovery from peripheral nerve injury
Source: Front Physiol. 2025 Oct 21;16:1653032. doi: 10.3389/fphys.2025.1653032 (PMC12584819; doi:10.3389/fphys.2025.1653032)
Supplement: Supplementary file 1 [file DataSheet1.pdf]

Title: Resistance Exercise Promotes Functional Recovery from Peripheral Nerve Injury

Authors: Hameed Al-Sarraf*, Hind Al Mallah, and Abdeslam Mouihate

Department of Physiology, College of Medicine, Kuwait University, PO Box 24923, Safat-13110, Kuwait

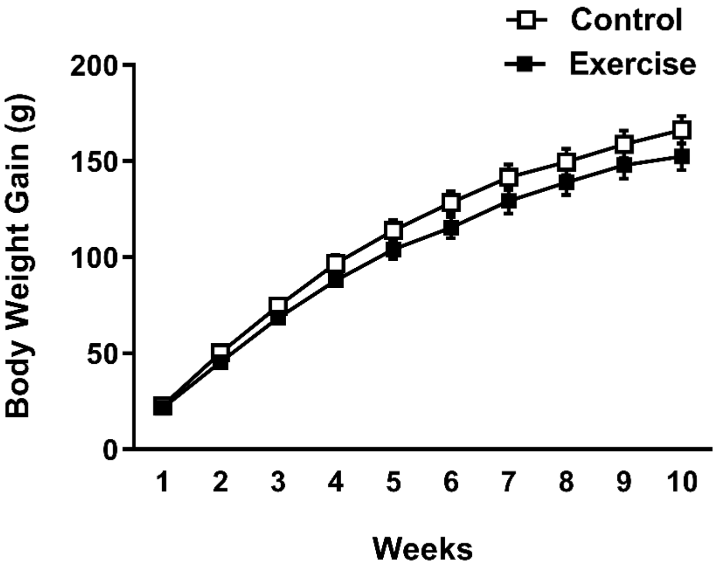

Supplementary Data: Body weight gain.

The body weight gains were measured weekly in control and resistance-exercised rats. Data are presented as mean ± SEM (n = 30 rats/group). Resistance exercise did not significantly affect body weight gain. ($p > 0.05$).
